# Comparative effectiveness of two popular weight loss programs in women III: health and fitness markers

**DOI:** 10.1186/1550-2783-8-S1-P5

**Published:** 2011-11-07

**Authors:** Jonathan Oliver, Michelle Mardock, Brittanie Lockard, Mike Byrd, Sunday Simbo, Andrew Jagim, Julie Kresta, Claire Baetge, Peter Jung, Majid Koozehchian, Deepesch Khanna, Mike Greenwood, Chris Rasmussen, Richard Kreider

**Affiliations:** 1 Exercise & Sport Nutrition Lab. Texas A&M University, College Station, TX 77843, USA

## Background

A number of commercial diet and exercise programs are promoted to help people lose weight and improve fitness. However, few studies have compared the effects of following different types of exercise and diet interventions on weight loss and/or changes in health and fitness markers. The purpose of this study was to compare the efficacy of a more structured meal plan based diet intervention and supervised exercise program that included resistance-exercise to a traditional point based diet program with weekly counseling and encouragement to increase physical activity.

## Methods

Fifty-one sedentary women (35±8 yrs, 163±7 cm; 90±14 kg; 47±7% body fat, 34±5 kg/m^2^) were randomized to participate in the Curves (C) or Weight Watchers (W) weight loss programs for 16-wks. Participants in the C program were instructed to follow a 1,200 kcal/d diet for 1-week, 1,500 kcal/d diet for 3 weeks, and 2,000 kcals/d diet for 2-weeks consisting of 30% carbohydrate, 45% protein, and 30% fat. Subjects then repeated this diet. Subjects also participated in the Curves circuit style resistance training program 3 days/week and were encouraged to walk at brisk pace for 30-min on non-training days. This program involved performing 30-60 seconds of bi-directional hydraulic-based resistance-exercise on 13 machines interspersed with 30-60 seconds of low-impact callisthenic or Zumba dance exercise. Participants in the W group followed the W point-based diet program, received weekly counseling at a local W facility, and were encouraged to increase physical activity. Body mass index (BMI), waist and hip circumference; as well as changes in resting heart rate (RHR) and blood pressure (BP) were obtained at 0, 4, 10, & 16 wks and analyzed by multivariate analysis of variance (MANOVA) with repeated measures for changes. Measurements of strength and endurance were obtained at 0 and 16 weeks.

## Results

MANOVA analysis of anthropometry data revealed an overall Wilks’ Lamda significant time (p=0.001) and diet (p=0.05) effect with no significant time x diet effect (p=0.29). After 16 weeks both groups decreased BMI (C -2.5±1.9, -4.6±3.2, -5.1±3.7; W -3.1±1.5, -6.0±2.7, -7.1±4.7 %;p=0.10), waist circumference(C -2.8±3.7, -5.4±5.2, -6.2±5.1;W -1.1±5.6, -4.2±6.0, -5.9±5.5 %;p*_q_*=0.21) and hip circumference (C -1.7±2.1, -4.1±3.4, -4.7±4.0;W -1.5±3.3, -4.3±3.2, -6.2±4.1 %;p*_q_*=0.15) over time; with no differences seen between groups. MANOVA analysis of RHR and BP data revealed an overall Wilks’ Lambda significant time (p=0.008) effect with no diet (p=0.71) or time x diet (0.11) effect. Both groups significantly decreased RHR (C -5.6±13.2, -7.4±13.8, -0.7±11.3;W -5.9±18.1, 0.2±20.9, -0.9±20.9 %;p*_q_*=0.22), systolic BP (C -2.4±6.5, -2.9±9.3, -3.8±8.8;W -4.3±8.6, -3.5±10.1, -4.1±7.5 %;p*_q_*=0.53), and diastolic BP (C -5.1±10.4, -1.5±13.0, -1.6±13.0;W -5.1±11.4, -6.4±11.6, -5.7±10.0 %;p=0.11) over time; with no differences seen between groups. MANOVA analysis of strength and strength endurance revealed a significant difference between groups (p=0.008) participants in C improved their leg press 1RM (C 5.6±16; W 0.0±19%), bench press 1RM (C 4.5±15; W -0.9±10 %), leg press endurance (C 22.3±85; W 7.1±54 %), and bench press endurance (C 45.4±97; W -10.5±39%) to a greater degree. No significant difference were seen in changes in peak oxygen uptake (C 11.1±11.5; W 9.3±8.5%;p=0.52).

## Conclusion

Results indicate that participation in C and W programs improved several markers of health and fitness. However, adherence to a more structured meal plan based diet combined with a supervised exercise program promoted more favorable changes in strength and endurance.

## Funding

Supported by Curves International (Waco, TX)

